# Electroacupuncture attenuates chronic fibromyalgia pain through the phosphorylated phosphoinositide 3-kinase signaling pathway in the mouse brain

**DOI:** 10.22038/ijbms.2019.35887.8547

**Published:** 2019-09

**Authors:** Chao-Tsung Chen, Jaung-Geng Lin, Chun-Ping Huang, Yi-Wen Lin

**Affiliations:** 1 Center for General Education, Chung Yuan Christian University, Taoyuan 32023, Taiwan; 2 College of Chinese Medicine, School of Chinese Medicine, China Medical University, Taichung 40402, Taiwan; 3 College of Chinese Medicine, Graduate Institute of Acupuncture Science, China Medical University, Taichung 40402, Taiwan

**Keywords:** Dorsal root ganglion, Electroacupuncture, Fibromyalgia, Phosphatidylinositol, Somatosensory cortex, Thalamus, 3-kinase

## Abstract

**Objective(s):**

Fibromyalgia (FM) is a central nervous system disorder characterized by widespread mechanical hyperalgesia due to unknown mechanisms. Several inflammatory mediators, such as interleukin-1 (IL-1), IL-6, IL-8, and tumor necrosis factor, are increased in the serum of FM patients. Although medications including pregabalin, duloxetine, and milnacipran are used to treat FM, the results are unsatisfying. In the present study we assessed whether electroacupuncture (EA) can reduce chronic FM pain and then proposed an underlying mechanism for this effect.

**Materials and Methods:**

Chronic FM pain was induced in mice by dual acid saline injection lasting up to 4 weeks.

**Results:**

Chronic FM pain was treated by EA manipulation, but not in the sham operated group. Phosphorylated phosphatidylinositol 3-kinase (pPI3K), protein kinase B, mechanistic target of rapamycin, and nuclear factor kappa-light-chain-enhancer of activated B cells were unaltered in the mouse dorsal root ganglion (DRG) and spinal cord (SC) after inducing FM and administering EA treatment. The pPI3K-associated nociceptive signaling pathway was increased in the thalamus of FM mice, but reversed by EA. Similar results were observed in the mouse somatosensory cortex.

**Conclusion:**

These data suggest that EA has a significant effect on a signaling pathway in brain areas of FM mice. These findings suggest the value of EA for clinical practice.

## Introduction

Fibromyalgia (FM) is a disabling disease that manifests as chronic, widespread nociceptive sensations. Patients experience spreading pain accompanied by fatigue, depression, memory problems, anxiety, sleep disturbances, and headaches. These symptoms impair the daily functioning of FM patients. The prevalence of FM is 2%–8% of the population (1, 2). For an unknown reason, FM occurs more frequently in females. FM is known to affect all ethnicities and ages, even presenting in children. Due to its chronic nature, FM requires long-term management. However, as the mechanism of action of FM is poorly understood, effective treatments are lacking. The pathogenesis of FM is puzzling and limited research is available to identify the etiology. Given that a chronic, long-term illness like FM can debilitate an individual, it is hoped that better understanding of the mechanism of FM can be used to develop more effective treatment strategies (3, 4).

Phosphoinositide 3-kinase (PI3K) belongs to a family of enzymes involved in cell growth, proliferation, and differentiation. PI3K can activate the AKT signaling pathway for cell proliferation and survival. The PI3K pathway is stimulated to protect astrocytes from apoptosis (5). PI3K can be subdivided into PI3Kα, PI3Kβ, PI3Kδ, and PI3Kγ. PI3Kα, PI3Kβ, and PI3Kδ which are all activated by receptor tyrosine kinases, which are transmembrane glycoproteins with enzymatic activity. In addition, PI3Kγ, can be activated by both G-protein-coupled receptors and Ras (6). Activation of PI3Ks can convert phosphatidylinositol 4,5-biphosphate (PIP2) into phosphatidylinositol 3,4,5-triphosphate (PIP3) to phosphorylate Akt (7, 8). This activation will then turn on the mTOR signaling pathway and further activate NFκB for transcription (9).

Acupuncture is a traditional technique that has been used for over 3,000 years to treat conditions such as stroke (10), dementia (11), and pain (12-15). Analgesia by acupuncture is generally accepted worldwide. The first study to demonstrate the analgesic effect of acupuncture was published in 1973 (16). More recent studies have shown that the analgesic effect of acupuncture is mediated by the release of endogenous opiates (17) and serotonin (18). Endogenous opiate concentrations in plasma have been shown to increase in response to acupuncture (19) or increase as neurotransmitters in the cerebrospinal fluid (20).

Our rationale for the current study is that electroacupuncture (EA) is effective for treating FM pain with unclear mechanisms. We hypothesized that EA could effectively treat mice with chronic FM-associated mechanical hyperalgesia by regulating the Phosphoinositide 3-kinase (PI3K) pathway. The PI3K signaling pathway is involved in a mouse inflammatory pain model (12-15). Accordingly, we aimed to identify whether EA attenuated mechanical hyperalgesia in a mouse model of chronic FM. We also verified whether the PI3K signaling pathway was involved in the peripheral and central nervous systems.

## Materials and Methods

***Animals***

We used 8–12 week old C57BL/6 mice purchased from BioLASCO Co Ltd (Taipei, Taiwan) for all experiments. The mice were randomly assigned to four groups (n= 8 per group): (1) Normal (controls), (2) FM, (3) FM+2 Hz EA, and (4) FM+sham EA. The sample size required for an alpha of 0.05 and a power of 80% was six animals per group. Mice were housed in a room under a 12/12 hr light/dark cycle with *ad libitum* access to water and food. All procedures were approved by the Institutional Animal Care and Use Committee of China Medical University (No. 2018-110) and conducted in accordance with the Guide for the Use of Laboratory Animals provided by the National Research Council and the ethical guidelines of the International Association for the Study of Pain. The number of animals used and their suffering were minimized.

**Figure 1:**
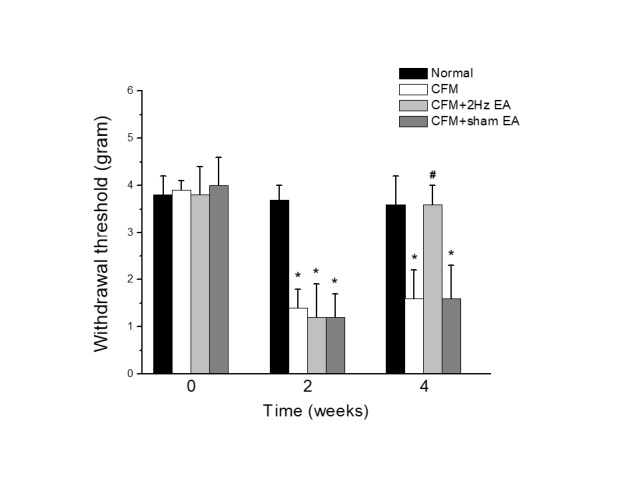
Mechanical withdrawal thresholds in each group of mice. Normal saline injection (Normal group, n=8), CFM (Acid saline-induced FM pain), 2 Hz EA (Acid saline-induced FM pain treated with 2 Hz EA), and sham EA (Acid saline-induced FM pain treated with sham EA). *P<0.05 vs. Normal group. #*P*< 0.05 vs. CFM group

**Figure 2:**
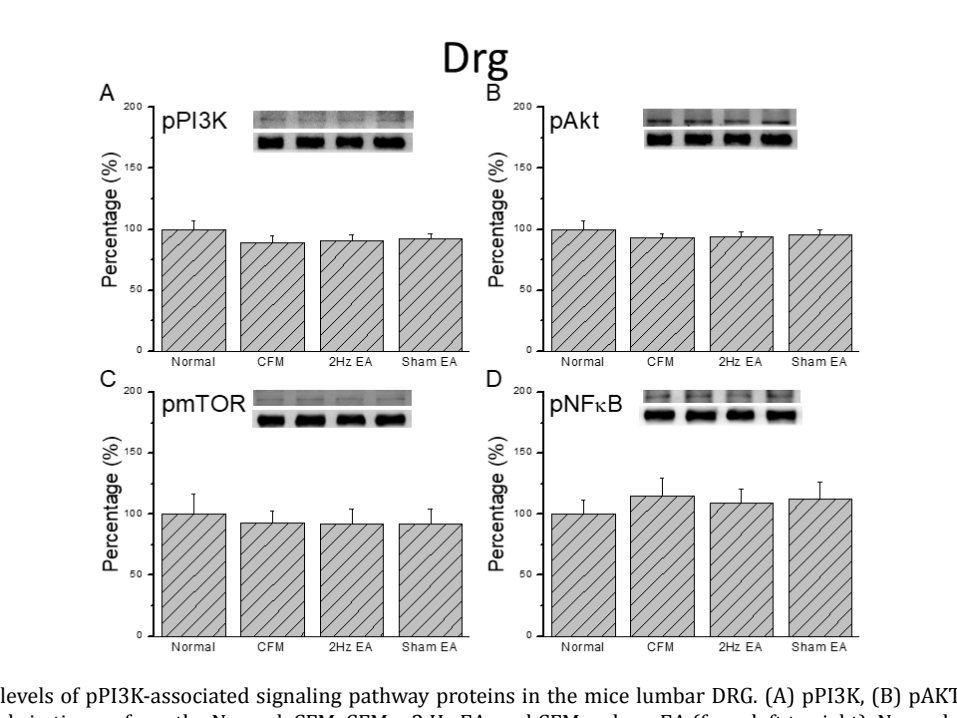
Expression levels of pPI3K-associated signaling pathway proteins in the mice lumbar DRG. (A) pPI3K, (B) pAKT, (C) pmTOR, and (D) pNFκB expression levels in tissues from the Normal, CFM, CFM + 2 Hz EA, and CFM + sham EA (from left to right). Normal = normal mice; CFM = chronic fibromyalgia mice; 2Hz EA = CFM + 2 Hz EA. Sham EA = CFM + sham EA. *P<0.05 compared with the normal group. #*P*<0.05 vs. CFM group. The Western blot bands at the top show the target protein. The lower bands are internal controls (β-actin or α-tubulin)

**Figure 3:**
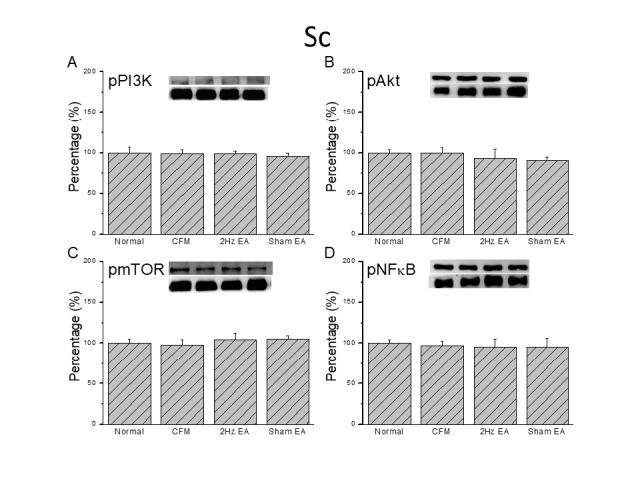
Expression levels of pPI3K-associated signaling pathway proteins in the mice lumbar SC. (A) pPI3K, (B) pAKT, (C) pmTOR, and (D) pNFκB expression levels in tissues from the Normal, CFM, CFM + 2 Hz EA, and CFM + sham EA (from left to right). Normal = normal mice; CFM = chronic fibromyalgia mice; 2Hz EA = CFM + 2 Hz EA. Sham EA = CFM + sham EA. *P<0.05 compared with the normal group. #*P*<0.05 vs. CFM group. The western blot bands at the top show the target protein. The lower bands are internal controls (β-actin or α-tubulin)

**Figure 4:**
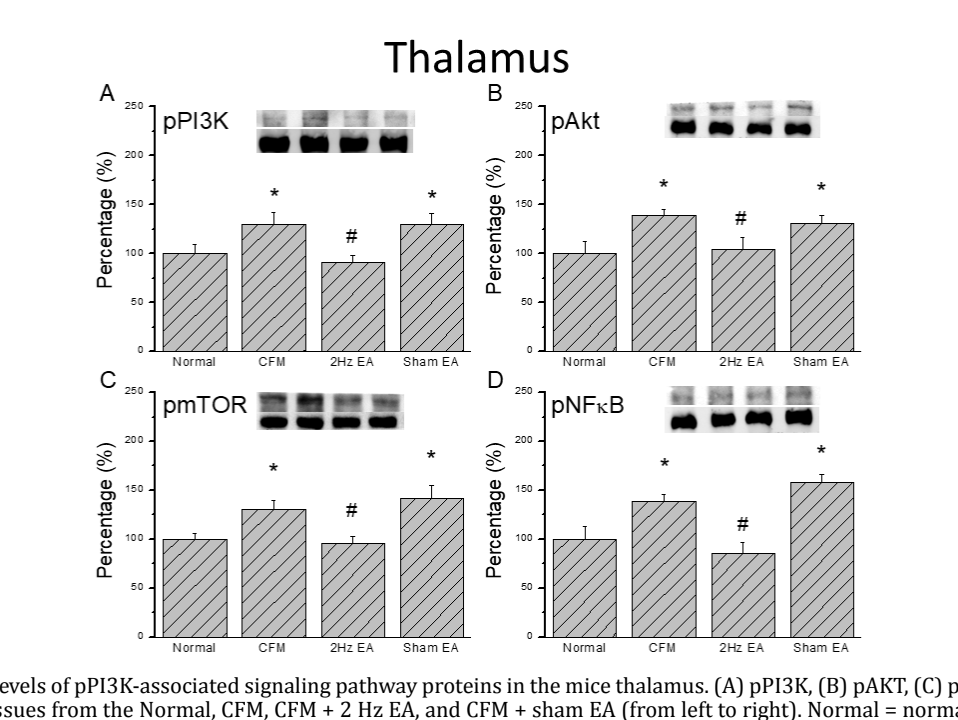
Expression levels of pPI3K-associated signaling pathway proteins in the mice thalamus. (A) pPI3K, (B) pAKT, (C) pmTOR, and (D) pNFκB expression levels in tissues from the Normal, CFM, CFM + 2 Hz EA, and CFM + sham EA (from left to right). Normal = normal mice; CFM = chronic fibromyalgia mice; 2Hz EA = CFM + 2 Hz EA. Sham EA = CFM + sham EA. *P<0.05 compared with the normal group. #*P*<0.05 vs. CFM group. The Western blot bands at the top show the target protein. The lower bands are internal controls (β-actin or α-tubulin)

**Figure 5:**
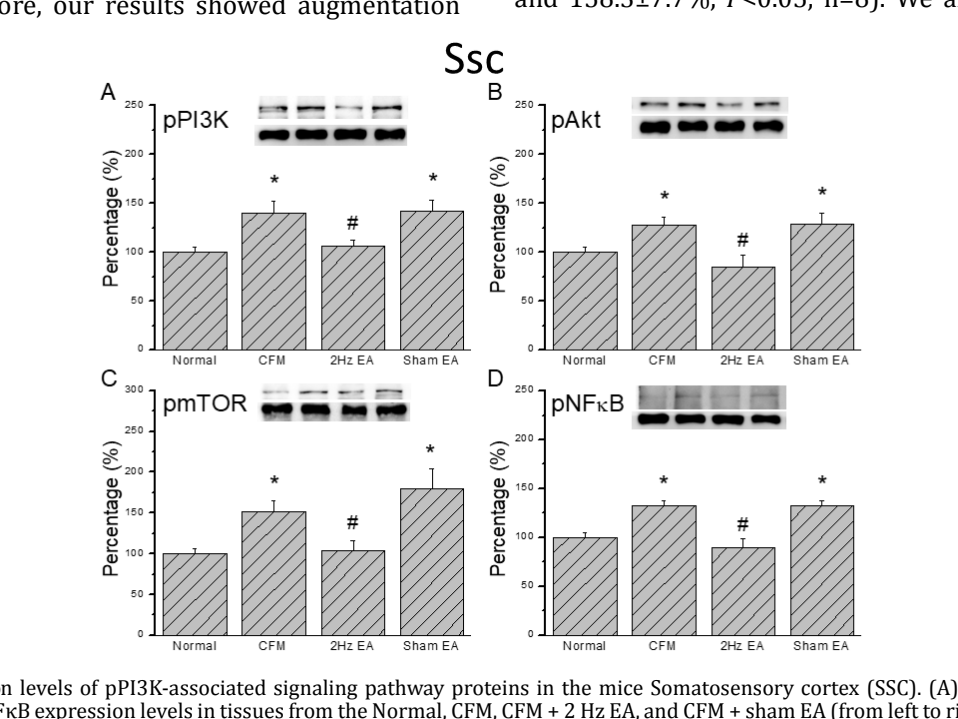
Expression levels of pPI3K-associated signaling pathway proteins in the mice Somatosensory cortex (SSC). (A) pPI3K, (B) pAKT, (C) pmTOR, and (D) pNFκB expression levels in tissues from the Normal, CFM, CFM + 2 Hz EA, and CFM + sham EA (from left to right). Normal= normal mice; CFM= chronic fibromyalgia mice; 2Hz EA = CFM + 2 Hz EA. Sham EA = CFM + sham EA. *P<0.05 compared with the normal group. #*P*<0.05 vs CFM group. The Western blot bands at the top show the target protein. The lower bands are internal controls (β-actin or α-tubulin)

***FM induction and mechanical hyperalgesia measurement***

All mice except controls received a 20 μl injection of acidic saline (pH 4.0) into the right gastrocnemius muscle (IM) under isoflurane (1%) anesthesia on day 0. The second acidic saline injection was administered on day 5 to establish the FM mouse model. The acidic saline was prepared in 10 mM 2-[N-morpholino]ethanesulfonic acid and further adjusted to pH 4.0 with 1 N NaOH. Mechanical sensitivity was tested at weeks 2 and 4 after the first acidic saline injection. Mechanical sensitivity was measured by testing the strength of responses to stimulation with three applications of electronic von Frey filaments (North Coast Medical, Gilroy, CA, USA). Mice were placed on a metal mesh and adapted to the new environment for at least 30 min before testing.

***Acupuncture manipulation***

EA was administered in the morning (9:00–10:00 am) starting 2 weeks after the induction of FM. EA treatment lasted for 15 min at a frequency of 2 Hz and an amplitude of 1 mA and was performed three times per week for 2 weeks. EA was delivered using stainless steel needles (0.5 inch, 32 G, Yukuang, Taipei, Taiwan) vertically inserted into the muscle layer at a depth of 3–5 mm in the ST36 acupoint under 1% isoflurane anesthesia. The ST36 acupuncture point is located on the tibialis anterior muscle, approximately 1/6 of the distance from the patella to the lateral malleolus. In the FM + sham EA group, a needle was inserted into the ST36 acupoint without any rotation or twisting.

***Tissue sampling and Western blot analysis***

After the treatment period, mice were sacrificed and the lumbar dorsal root ganglion (DRG), spinal cord (SC), full thalamus, and somatosensory cortex (SSC) neurons were immediately excised for protein extraction. Total proteins were prepared by homogenizing the tissues in lysis buffer containing 50 mM Tris-HCl (pH 7.4), 250 mM NaCl, 1% NP-40, 5 mM EDTA, 50 mM NaF, 1 mM Na_3_VO_4_, 0.02% NaNO_3_, and 1× protease inhibitor cocktail (Amresco, Solon, OH, USA). The extracted proteins (30 μg per sample, according to the BCA protein assay) were subjected to 8% sodium dodecyl sulfate-Tris glycine gel electrophoresis and transferred to a PVDF membrane. The membrane was blocked with 5% non-fat milk in TBS-T buffer (10 mM Tris pH 7.5, 100 mM NaCl, 0.1% Tween 20), incubated with primary antibodies against pPI3K (~125 kDa, 1:1000, Millipore, Bedford, MA, USA), pAkt (~60 kDa, 1:1000, Millipore), pmTOR (~60 kDa, 1:500, Millipore), and pNFκB (~65 kDa, 1:1000, Millipore) in TBS-T and 1% bovine serum albumin, and incubated for 1 hr at room temperature. A peroxidase-conjugated anti-rabbit antibody (1:5,000) was used as the secondary antibody. The bands were visualized using an enhanced chemiluminescent substrate kit (Pierce, Rockford, IL, USA) with LAS-3000 Fujifilm (Fuji Photo Film Co Ltd, Tokyo, Japan). Where appropriate, the image intensities of specific bands were quantified using NIH ImageJ software (Bethesda, MD, USA). The protein ratios were determined by dividing the target protein intensities by the intensity of α-tubulin or β-actin in the same sample. The calculated ratios were adjusted by dividing the ratios from the same comparative group relative to controls.

***Statistical analysis***

All data are expressed as mean±standard error. Differences between the normal, FM, 2Hz EA, and sham EA groups were tested by analysis of variance followed by a *Post hoc* Tukey’s test. The level of statistical significance was *P*<0.05.

## Results

***Induction of chronic FM pain and further attenuation by a 2 Hz EA manipulation, but not sham treatment***

A similar mechanical threshold was observed in the normal, FM, 2 Hz EA, and sham operated groups under basal conditions (Figure 1). Significant mechanical hyperalgesia was observed in the FM, 2 Hz EA, and sham EA groups two weeks after inducing FM. Mechanical hyperalgesia was maintained in the FM mice during week 4. In addition, 2 Hz EA treatment reliably attenuated mechanical hyperalgesia. Attenuation was not observed in the sham EA group, suggesting the specificity of EA.

***Chronic FM pain did not alter expression of the PI3K signaling pathway in the mouse peripheral DRG or central SC***

Western blot was used to quantify PI3K protein levels and sequential molecules in the mouse DRG. We confirmed the presence of pPI3K in normal mouse DRG (Figure 2A, 100.1±7.2%, *P*>0.05, n=8). Chronic FM induction did not alter expression (Figure 2A, 88.5±6.5%, *P*>0.05, n=8), and potentiation of pPI3K was not altered by 2 Hz or sham EA (Figure 2A, 90.2± 5.2% and 92.3±3.8%, *P*>0.05, n=8). We then tested if downstream pAkt shared similar mechanisms with pPI3K. The results showed that pAkt was unchanged in chronic FM mice (Figure 2B, 93.3±3.2%, *P*>0.05, n=8). Similarly, expression was similar in the 2 Hz EA and sham control groups (Figure 2B, 93.6±4.2% and 95.8± 3.6%, *P*>0.05, n=8). Next, we quantified the expression of pmTOR, a downstream pAkt factor, in the mouse DRG. We found that pmTOR was unchanged in chronic FM mice (Figure 2C, 92.6±10.2%, *P*>0.05, n = 8), and that expression was similar in the 2 Hz EA and sham control groups (Figure 2C, 92.1±12.1% and 92.2±12.4%, *P*>0.05, n= 8). Furthermore, pNFκB was unaltered in FM mice (Figure 2D, 114.9±14.8%, *P*>0.05, n=8), and expression was similar in the 2 Hz EA and sham control groups (Figure 2D, 109.3±11.2% and 112.5±13.7%, *P*>0.05, n= 8). We also determined that PI3K, pAkt, pmTOR, and pNFκB were unchanged in the SC of chronic FM mice (Figure 3).

***Chronic FM pain increases expression of the PI3K signaling pathway in the mouse central thalamus and SSC***

We then examined PI3K protein expression in the central thalamus to determine if the PI3K signaling pathway is involved in central sensitization of chronic FM pain. Our data confirmed the presence of PI3K in the mouse thalamus and showed that the level was increased in the chronic FM group (Figure 4A, 100.1±9.2% and 129.6 ± 12.3%, *P*<0.05, n=8). Potentiation was reversed by the 2 Hz EA stimulation, but not in the sham operated group (Figure 4A, 90.9±7.4% and 129.6±11.7%, *P*<0.05, n=8). Furthermore, our results showed augmentation of pAkt in the thalamus of FM mice (Figure 4B, 138.4 ± 6.7%, *P*<0.05, n=8), which was attenuated by 2 Hz EA in FM mice (Figure 4B, 104.2±12.6%, *P*<0.05, n=8) but not in the sham control group (Figure 4B, 131.1±7.7%, *P*<0.05, n=8). Similar results were observed for pmTOR level in the FM (Figure 4C, 130.3±9.4%, *P*<0.05, n=8), EA (Figure 4C, 95.3±7.2%, *P*<0.05, n=8), and sham EA groups (Figure 4C, 141.8±12.8%, *P*<0.05, n=8). To identify transcriptional regulation of FM and EA, we further verified NFκB expression in the mouse thalamus. We found that pNFκB was expressed in the mouse thalamus and increased in FM mice (Figure 4D, 138.6±6.9%, *P*<0.05, n=8). This increase was reversed by 2 Hz EA, but not by sham EA (Figure 4A, 84.9±11.5% and 158.3±7.7%, *P*<0.05, n=8). We also examined the SSC, which is sensitive to pain, to assess whether the PI3K signaling pathway participates in this brain area. The data confirmed that pPI3K is present in the mouse SSC and is significantly increased in FM mice (Figure 5A, 140.3±11.2%, *P*<0.05, n=8). Again, augmentation was attenuated by 2 Hz EA, but not by sham EA (Figure 5A, 105.9±6.4% and 141.5±11.9%, *P*<0.05, n=8). We also found that pAkt was increased in the SSC of FM mice and could be reduced by 2 Hz EA (Figure 4A, 127.1±9.3% and 85.1±11.8%, *P*<0.05, n=8), but that there was no effect in the sham group (Figure 4A, 128.3±11.9%, *P*< 0.05, n=6). Similar results were observed for pmTOR and pNFκB protein expression in the mouse SSC (Figure 5C, D).

## Discussion

FM pain is strongly associated with inflammation. As demonstrated by clinical study, inflammatory mediators like interleukin (IL)-1β, IL-6, IL18, and tumor necrosis factor (TNF)α are commonly elevated in FM patients (21, 22). Ohgidani *et al*. reported that microglia are hypersensitive to ATP in patients with FM; TNF-α secreted from microglia appears to play a crucial role in the development of FM (23). A recent study suggested that the cyclooxygenase-2 and PI3K/Akt signaling pathways are crucial in chemotherapy-induced neuropathic pain (24). Local inflammation initiates significant central sensitization of nociceptive SC neurons, and TNF and AMPA receptors are known to be involved in this process. Pre-treatment with a PI3K inhibitor can reliably reduce potentiation of the AMPA receptor due to inflammation (25). Liu *et al*. reported that the PI3K-Akt signaling pathway was significantly increased in the SC of chronic constriction injury (CCI) rats. CCI also potentiates phosphorylation of the AMPA receptor and induces mechanic allodynia (26). The results of the present study indicated that pPI3K-pAkt was unaltered in the peripheral DRG and central SC neurons in all groups. The increase in pPI3K-pAkt in the mouse thalamus and SSC after inducing FM could be reversed by 2 Hz EA, but there was no effect in the sham group. This is the first report demonstrating that pPI3K-pAkt is involved in chronic FM pain.

A recent study of CCI rats showed that the pain threshold decreased on days 7 and 14 after induction, whereas both mRNA and protein levels of PI3K, Akt, and mTOR increased (27). Furthermore, glial fibrillary acidic protein, which is used to mark OX-42, simultaneously increased in the CCI group. Pritchard *et al*. reported that blocking PI3K-γ reliably attenuated inflammatory carrageenan-induced hyperalgesia and c-Fos expression in the rat SC (28). Taken together, these results suggest that pPI3K-pAkt plays a crucial role in the maintenance of chronic FM pain. 

Our previous study showed that mechanical hyperalgesia was induced by dual acidic injections in FM mice model. Nociceptive receptors, including ASIC3, Nav1.7, and Nav1.8, were also increased in the DRG and SC in this FM mouse model. An increase in pPI3K was also observed in the central thalamus of FM mice, highlighting its crucial role in FM (13). A recent study demonstrated that mTOR is a direct target of miR-183, which can modulate the vascular endothelial growth factor to attenuate neuropathic pain. Inhibition of mTOR by upregulation of miR-183 has also been shown to greatly relieve neuropathic pain (29). Kwon *et al*. reported that rapamycin, a mTOR inhibitor, significantly attenuates mechanical allodynia. They also indicated that rapamycin reduces postsynaptic density protein 95 (PSD95) and neural excitability in the insular cortex, suggesting that mTOR signaling plays a crucial role in modulating neuropathic pain (30). Chen *et al*. reported that an intrathecal injection of protein kinase C blocker prevents the development of acidic saline-induced chronic pain, and further demonstrated that mTOR-dependent protein synthesis is required to establish priming (31).

## Conclusion

In the present study, we first demonstrated the ability of an acidic saline injection to induce FM pain in a murine model. We then showed that mechanical hyperalgesia was attenuated by 2 Hz EA simulation, but not by sham. Notably, pPI3K, pAkt, pmTOR, and pNFκB were unaltered in the DRG and SC of chronic FM mice. These molecules were also unaltered after 2 Hz or sham EA treatments. By contrast, pPI3K, pAkt, pmTOR, and pNFκB were increased in the mouse thalamus and SSC after inducing FM. The increase in signals was attenuated by 2 Hz EA, but not by the sham EA. Taken together, these results suggest that chronic FM induces significant mechanical hyperalgesia accompanied by increased expression of the PI3K signaling pathway in the mouse thalamus and SSC. These findings support the potential application of EA for managing FM pain.
